# The Potential of Spot Size Control in Shaping the Thickness Distribution in Ultrashort Laser Deposition

**DOI:** 10.3390/ma17112712

**Published:** 2024-06-03

**Authors:** Antonella Lorusso, László Égerházi, Sándor Szatmári, Tamás Szörényi

**Affiliations:** 1 Department of Mathematics and Physics “E. De Giorgi”, University of Salento, 73100 Lecce, Italy; antonella.lorusso@unisalento.it; 2 Department of Medical Physics and Informatics, Albert Szent-Györgyi Medical School, University of Szeged, H-6720 Szeged, Hungary; egerhazi.laszlo@med.u-szeged.hu; 3 Institute of Physics, University of Szeged, H-6720 Szeged, Hungary; szatmaris@titan.physx.u-szeged.hu; 4 Department of Optics and Quantum Electronics, University of Szeged, H-6720 Szeged, Hungary

**Keywords:** sub-ps PLD, copper thin films, laser spot size, forward-peaked, femtosecond plasma

## Abstract

The availability of new-generation femtosecond lasers capable of delivering pulses with energies in the hundreds of mJ, or even in the joules range, has called for a revision of the effect of scaling spot size on the material distribution within the plasma plume. Employing a state-of-the-art Szatmári-type hybrid dye-excimer laser system emitting 248 nm pulses with a maximum energy of 20 mJ and duration of 600 fs, copper films were grown in the classical pulsed laser deposition geometry. The exceptionally clean temporal profile of the laser pulses yielded a femtosecond component of 4.18 ± 0.19 mJ, accompanied by a 0.22 ± 0.01 mJ ASE pedestal on the target surface. While varying the spot sizes, the plasma plume consistently exhibited an extremely forward-peaked distribution. Deposition rates, defined as peak thickness per number of pulses, ranged from 0.030 to 0.114 nm/pulse, with a gradual narrowing of the thickness distribution as the spot area increased from 0.085 to 1.01 mm^2^ while keeping the pulse energy constant. The material distribution on the silicon substrates was characterized using the *f*(Θ) = *A*cos*^k^*Θ + (1 − *A*)cos*^p^*Θ formalism, revealing exponents characterizing the forward-peaked component of the thickness profile of the film material along the axes, ranging from *k* = 15 up to exceptionally high values exceeding 50, as the spot area increased. Consequently, spot size control and outstanding beam quality ensured that majority of the ablated material was confined to the central region of the plume, indicating the potential of PLD (pulsed laser deposition) for highly efficient localized deposition of exotic materials.

## 1. Introduction

Pulsed laser deposition, PLD, is acknowledged as a highly versatile technique for thin film fabrication. Through numerous reports, it has been demonstrated that PLD harnesses the power of highly non-equilibrium processes, initiated by the temporally and spatially concentrated energy input, to convert virtually any material into a plasma state. This plasma is then used to grow thin films by capturing its components onto substrates positioned in the path of the expanding plasma cloud. A distinguishing characteristic of PLD is the strongly forward-peaked material flow, which—in the simplest case of a static target–substrate geometry—results in laterally non-homogeneous film growth.

This is often viewed as a drawback within the thin film community, limiting the competitiveness of PLD applications. In this study, we demonstrate that the thickness distribution of PLD films can be effectively controlled and thereby optimized for specific applications.

### 1.1. Nanosecond Pulsed Laser Deposition

The spatial distribution of the plasma components and the resulting thickness distribution of the film are influenced by several factors. These include the material to be ablated, the energy and duration of the laser pulses, as well as the size and shape of the irradiated area. While the overall tendencies and their extent caused by changes in the process parameters remain consistent across different target materials, the specific impact of the individual process parameters on the material distribution within the plume can vary significantly.

In the PLD literature, the energy density or fluence, defined as the ratio of the pulse energy to the irradiated area, stands out as a fundamental process parameter. As a quotient, this parameter can be adjusted either by altering the pulse energy while keeping the spot area constant or by changing the dimensions of the irradiated area while maintaining a constant pulse energy. To control fluence, the prevalent practice is tuning pulse energy while keeping the spot size fixed, primarily due to the ease of changing and measuring pulse energy compared to realigning beam paths and measuring processed area dimensions precisely. Notably, when comparing literature data involving different spot sizes, distinguishing the effects of energy or spot size changes is challenging. Nevertheless, there is a consensus that the maximum film thickness in the central region generally increases with the fluence, although in a nonlinear fashion [1,2,3].

Model predictions regarding the polar distribution of the emitted material as a function of fluence show inconsistency. Kools suggested that in the absence of plasma effects, particularly at low fluences, the distribution should remain independent of fluence [4]. On the other hand, Gorbunov and Konov predicted a slight broadening [5], while the majority of the authors expected narrowing [1,3,6,7]. Experimental results generally support this latter assumption, with only a few exceptions. Santagata et al. reported no significant change in the thickness distribution of the films with *n* = 3.8 ± 0.6 in the *f*(Θ) = cos*^n^*Θ formalism at fluences ranging from 0.1 to 10 Jcm^−2^ [8]. In contrast, Mele et al. observed a decrease in the exponent from nine to five when reducing the fluence from 12 Jcm^−2^ to 6 Jcm^−2^ [9]. Singh consistently observed more spread-out distributions at low fluences and more forward-directed distributions as the fluence increased when ablating with a XeCl excimer laser at 308 nm. The films exhibited a cos^8–12^Θ thickness distribution.

The fundamental assertion of Thum-Jager and Rohr was that the angular distribution of both neutrals and ions broadens as fluence decreases, while keeping the spot size constant [3,9,10,11,12,13], with ions exhibiting a narrower angular distribution [14], consistent with the findings of Doggett and Lunney [15]. Thum-Jager and Rohr noted that the distribution of ionic components could be described by a single cos*^n^*Θ term, whereas an additional component resulting in a form of *a*cos*^k^*Θ + *b*cos*^p^*Θ was necessary to account for the contributions of the neutrals. When ablating titanium, they observed a gradual narrowing of the distribution of the ionic component with *n* increasing from 6 up to 14 with an increase in charge number. In another series of experiments, they derived a nearly linear increase in the exponent with atomic mass. Based on these observations, the authors explained the energy (or fluence) dependency of the angular distribution of a particle flux consisting of both neutrals and ions by changes in the ions-to-neutrals ratio. This approach also explains why the distributions can be fitted by two terms, with the steeper contribution (from ions) increasing with pulse energy up to *p* = 27, while the broader component (with exponents around or less than 10) pertains to neutrals [16]. Mann and Rohr formulated a general statement, asserting that for ions of charge number *z*, the angular distribution of the plume scales with cos^2*z*+1^ [17].

Konomi et al. investigated the relationship between the angular distribution of the deposited material and the atomic weight of metals [18]. In their two-component fitting, the exponents varied between 3 and 24 for different metals, with Ta exhibiting the highest value, consistent with [14,16,19]. Incidentally, using two-component fits is a common practice, especially in describing the thickness distribution [10,11,20,21,22,23].

Early studies investigating the experimental and theoretical aspects of plasma plume evolution, spanning from Knudsen layer formation through adiabatic expansion to reaching the substrate, revealed that the highest expansion velocities occurred along the direction of smallest dimensions and vice versa [9,24]. This observation, recurring in various forms across different models [1,4,25] explains why the forward-peaking nature of the plume becomes more pronounced with increasing spot dimensions, and why flip-over occurs for asymmetrical spots [9,24,26,27,28,29]. R. K. Singh emphasized in his fundamental work that “the laser-irradiated spot size is the most important parameter which controls the spatial thickness variations” [7]. Primarily drawing from excimer laser results, Singh concludes that for spot sizes up to approximately 100 µm in diameter, the plasma plume assumes a spherical shape, between 100 µm and several millimetres in diameter, there is a marked transition in shape, and by reaching 8 mm, the plume transforms into a one-dimensional form. Singh mentions film thickness distributions between cos^2.5^Θ and cos^12^Θ. S. S. Harilal supported these findings by illustrative fast photography images [30].

To investigate the effect of changing spot size experimentally, the following two approaches are commonly employed: (i) When the fluence is kept constant and the spot size is adjusted, the concomitant changes in energy and spot size unavoidably superimpose. Since increasing the spot size requires an increase in pulse energy, and both adjustments transform the shape of the plume in the same way, this approach masks the actual effect of changes in spot size. (ii) Experiments conducted with constant pulse energy more clearly demonstrate the effect of changing spot size. Therefore, despite the technical difficulties in maintaining consistent beam characteristics across a broad range of spot areas, this approach proves to be more advantageous for studying the effect of spot size variations.

There is a consensus in the literature that the flux of a material becomes more forward-peaked with increasing spot size, while maintaining constant fluence [4,6,25]. To assess the extent of the effect of spot size adjustment on the plume expansion, Mele et al. probed the plume generated by ablating copper with 248 nm excimer pulses of 12 Jcm^−2^ fluence using three-dimensional fast photography. They observed a linear increase in the exponent *n* in the cos*^n^*Θ formalism with an increase in one linear dimension of the rectangular spot, confirming that increasing the area leads to a more forward-peaking plume [9]. The results of Haverkamp et al. [31] indicated that even minor changes in the area caused observable variations, with the effect being more pronounced at higher fluences.

In an example of maintaining constant pulse energy while varying spot size, Figure 5 in [32] demonstrates that increasing the fluence (resulting from decreasing the spot size by a factor of 10) leads to a broader plume with a somewhat reduced ion flux at the peak of the distribution. The results of Tyunina et al. [33] suggest that while the shape of the spot is a decisive parameter, for areas in the mm^2^ order, the effect of the changes in the area is negligible. Weaver and Lewis investigated the impact of both spot size and fluence variations on the characteristics of copper films [12]. Their results suggest that adjusting the spot size was more effective in controlling the distribution than changing the fluence.

### 1.2. Femtosecond Pulsed Laser Deposition

The excitement surrounding femtosecond lasers in materials processing has been driven by the promise of clean ablation and the direct production of nanoparticles (NPs). While the fundamental trends observed in nanosecond pulsed laser deposition has remained largely unchanged in the fs regime, the coexistence of NPs with neutral and ionic components in the plasma generated by fs lasers has introduced additional complexity to the behavior of the expanding plume, particularly in terms of pulse energy and spot size.

The material distribution of the faster component of the fs plume, comprising ions and neutrals, was found to be much broader than the slower component characterized by a black-body-like continuous spectrum attributed to the emission of nanoparticles and/or large clusters. Through ICCD intensity contour plots of the plumes, De Bonis et al. derived exponents of *n* = 2.7 and *n* = 14.4 for the cos*^n^*Θ fit, describing the angular distribution of the primary particles and NPs, respectively [34]. These findings align with those of an Italian–Bulgarian collaboration [35], where ablation of nickel using 300 fs pulses at 527 nm and 0.8 Jcm^−2^ in high vacuum revealed a broader angular divergence of atomic species within the plume as compared to that of the nanoparticles.

It is widely accepted that increasing fluence results in a more forward-peaked ion plume [36,37], with variations in spot sizes influencing the extent of this phenomenon since irradiating a smaller area results in a broader plume. When ablating various metals, including copper, with 40 fs at 800 nm pulses focused to 100 µm in a broad fluence range spanning from 2.8 to 44 Jcm^−2^, Anoop et al. [38] demonstrated through ion dynamics analysis that the angular distribution of the ionic components progressively narrowed with increasing fluence in the 2.8 to 11.1 Jcm^−2^ domain and plateaued at larger fluences. This group made similar observations when ablating copper with 50 fs Ti–sapphire pulses, where the angular distribution of the copper ion flux gradually sharpened as the fluence was increased from 1.9 Jcm^−2^ to 4.7 Jcm^−2^ and finally to 10 Jcm^−2^ [36]. Anoop et al. additionally observed that within the entire range of approximately 3 to 25 times the ablation threshold fluence, the ion population exhibited a more forward-peaked distribution compared to the neutrals (atoms) in the plasma plume [39]. Notably, ion probe measurements at three pulse durations (50, 200, and 600 fs) and a fixed laser fluence (8.5 Jcm^−2^) indicated identical angular distributions, suggesting independence of the ion plume characteristics from pulse duration within the sub-picosecond domain.

The differences in the angular distribution of the three components of the fs plasma plumes significantly impact the thickness distribution of the deposited material as a function of the process parameters. In a study where Ni was ablated by 300 fs pulses at 527 nm on a fixed spot area of 6.09 × 10⁻⁴ cm^2^, the distribution of the deposited material, consisting of NPs as identified by the authors, was confined with increasing fluence from 0.14 to 0.63 Jcm^−2^ [40]. Conversely, a study conducted under very similar experimental conditions (approximately 250 fs pulses at 527 nm, focused on a spot area of 1.3 × 10⁻³ cm^2^) and investigating the angular variations of the deposited material (NPs) in the peak fluence range of 0.15 to 0.7 Jcm^−2^, revealed broadening of the distribution with increasing fluence in the 0.1 to 0.4 Jcm^−2^ domain, and with no further change up to 0.7 Jcm^−2^ [36]. Within the same fluence range, the ion flux was found to become more peaked at higher fluences [41,42]. When ablating silver with a laser system identical in wavelength and pulse duration to those used by us in the present study, focused on a rectangular spot of 0.62 mm × 0.26 mm = 0.16 mm^2^ area, Toftmann et al. [37] obtained similar differences in the angular distribution of the ions vs. film material, where the distribution of the ionic components was observed to be narrower. When comparing the angular distribution of the deposits and the ions for plumes generated with durations of 500 fs and 26 ns, both at 248 nm and using identical spot sizes, it was observed that the angular distribution of both the deposits and the ions was narrower in the case of fs pulses. In this aspect, researchers generally agree. If there is any apparent contradiction between the reported results, the reason for this may be the different contribution of the plume components, behaving differently with increasing fluence, to the growing film.

The way in which the spot dimensions influence the behaviour of the fs plume is consistent with what was observed in the ns case. While keeping the pulse energy constant at approximately 5 mJ while ablating an aluminium target with 40 fs, ~800 nm pulses, significant changes in the plume geometry were recorded by fast gated photography [43]. Increasing the spot diameter from 100 μm to 600 μm resulted in a transition of the nearly spherical plume to a highly collimated shape with cylindrical symmetry. Quantitatively, at 100 µm, the aspect ratio was approximately 1, indicating spherical symmetry, while at 700 µm, the aspect ratio rose to 3.7. These values remained consistent at later stages of the evolution. Moreover, the larger spot size correlated with narrower angular distribution of the ion flux, contrasting the significantly broader profile observed for the smaller spot size. Similar tendencies were described by Garrelie et al. for diamond-like carbon (DLC) films grown by ablating graphite with a Ti–sapphire laser (150 fs), while keeping the pulse energy fixed at 1.5 mJ [44], although the tendency was less pronounced due to the spot areas being smaller by one order of magnitude. The film thickness varied depending on the spot size and, due to the constant pulse energy, also on the fluence. At the smallest spot area of 2.9 × 10^−2^ mm^2^, a low growth rate of 20 nm/min was obtained with a broad thickness distribution. As the spot size increased, the growth rate also increased, reaching 50 nm/min at 0.11 mm^2^, accompanied by a narrowing of the thickness distribution. Similar findings regarding fluence were discussed by the same authors in [45].

The previous critical analysis of the relevant literature suggests that adjusting the dimensions of the ablated spot could significantly affect both the growth rate and the lateral distribution of the ablated material. For a constant spot size, decreasing the energy (and thereby the fluence) leads to a decrease in the central film thickness due to a lesser amount of material being ablated. Conversely, in the case of constant energy, the increase in central thickness is a consequence of changes in the shape of the plasma plume that accompany the increase in the spot size. When maintaining constant fluence, the two tendencies enhance each other, making it difficult to evaluate the efficacy of spot size variation, explaining why it is more reasonable to investigate this issue at constant energy. Under such circumstances, the spot size and the fluence are inversely proportional to each other, evoking competitive effects. Beside the fact that this relationship has hardly been investigated, especially recently, the relevance of this research lies in the fact that the availability of modern lasers with sufficiently large energy and beam diameter are capable of producing a large spot size. Our aim is to demonstrate the following: (i) spot size adjustment has been underrated in controlling the film thickness and distribution, (ii) there exists a spot size range where the reduction in material ablation rate caused by decreasing fluence is counterbalanced by the confinement effect resulting from the increased spot size, and (iii) at spot sizes large enough, a very strongly forward-peaked plasma plume is generated that allows the majority of ablated material to be confined to a columnar volume, giving rise to the deposition of the ablated species to a well-localized substrate area.

## 2. Materials and Methods

### 2.1. Materials

For this case study, copper was selected as the model material for ablation, since copper PLD had been extensively studied, facilitating comparison with the literature data. The oxygen-free polycrystalline copper rod, 1″ in diameter and with 99.99% purity, was purchased from the Goodfellow Cambridge Office (Huntingdon, UK). Copper targets with a thickness of 5 mm were obtained using a high-precision lathe machine. The targets were polished to reduce surface roughness and then sonicated in acetone for 10 min, followed by rinsing in isopropyl alcohol. During ablation, the target was rotated to prevent crater formation.

### 2.2. Experimental Procedure

During laser material processing or pulsed laser deposition, the maximum spot area that can be utilized is technically determined by the ablation threshold fluence of the given material and the maximum available pulse energy. The new generation Szatmári-type [46] hybrid dye-excimer laser system enabled 248 nm pulses with over 20 mJ maximum energy and 600 fs pulse duration through amplification in a twin-tube amplifier module with a two-pass amplification geometry in each tube. This allowed for a rather broad adjustment of the spot size, covering the critical diameter domain ranging from hundreds to thousands of micrometres [43].

When employing ultrashort pulse lasers in materials processing, special attention should be paid to ensuring the temporal cleanliness of the pulse. A peculiarity of the laser system used in this study is the absence of ps pre-pulses due to the direct amplification system. The special amplification geometry, optimized for high energy contrast, in conjunction with spatial filtering between the first and second passes [47], ensured not only a notable femtosecond to nanosecond energy ratio, E_fs_/E_ASE_, of 19:1 for this series of experiments but also homogeneous energy distribution across the entire cross-section of the beam. These two characteristics created the experimental conditions optimized for investigating the effect of spot size variation on the thickness distribution.

The beam was focused by a Suprasil lens of *f*/10 at 45° angle onto the 1″ diameter target rotating in an HV chamber with a base pressure of 10^−4^ Pa. Serving as a diaphragm for the initially rectangular beam, the 2″ diameter lens generated an elliptical laser spot on the target surface. The size of the laser impact area was adjusted by translating the focusing lens along the optical axis, ensuring that the focal point always remained within the target. The beam cross-sections on the entrance window and the protective quartz plate behind, located approx. 100 mm from the lens of 500 mm focus distance remained practically the same when changing the lens position, guaranteeing no difference in the energy losses. To maintain stable operation during the experiments, the laser output was set at 20 mJ. The energy losses led to 4.4 ± 0.2 mJ on the target surface. Based on the 19:1 energy contrast, this corresponded to E_fs_ = 4.18 ± 0.19 mJ and E_ASE_ = 0.22 ± 0.01 mJ. Throughout the experiments, this fixed pulse energy was used while varying the spot size.

Since preliminary experiments indicated that several thousand pulses resulted in films with a few hundreds of nanometres in maximum film thicknesses at this pulse energy, all samples were deposited with a total of 10,000 laser pulses at a repetition rate of 2 Hz.

Due to the difference in their divergence, the femtosecond and the ASE components of the pulses produced different spot sizes on the target surface. The homogeneous energy distribution of the beam facilitated precise delineation of the irradiated regions, allowing for accurate determination of the areas. The imprint of the ASE component was discerned on UV photopaper of logarithmic response, appearing as a lighter halo extending over an area of 0.30 to 3.30 mm^2^, overlaying the smaller, darker mark left by the femtosecond component. The ratios of the femtosecond to ASE irradiated areas aligned with those obtained from far-field focus diagnostics.

**Figure 1:**
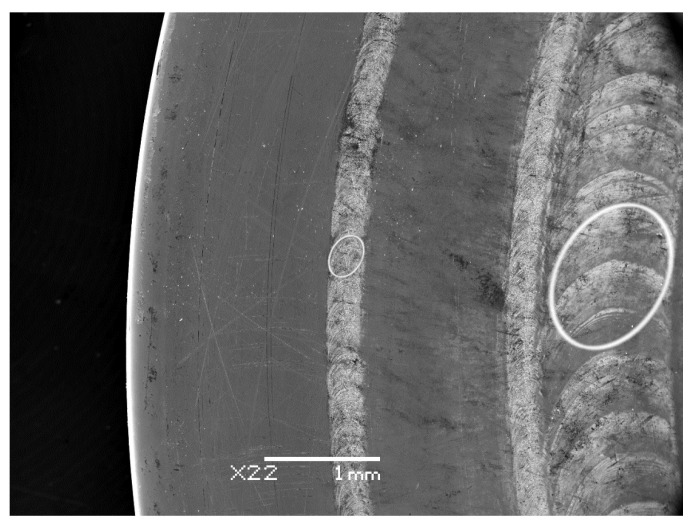
Low magnification SEM image of three tracks with the contours of two spots highlighted with an area of 1.01 mm^2^ (on the **right**) and 0.085 mm^2^ (on the **left**).

Spot sizes ranging from 0.085 to 1.01 mm^2^ were derived from low-magnification images taken from the processed Cu target surface as exemplified in Figure 1.

### 2.3. Sample Characterization

A JEOL JSM-6480 LV scanning electron microscope (JEOL Ltd., Welwyn Garden City, UK) was used to take low-magnification scans of the Cu target surface to determine spot sizes. To achieve a direct and reliable measurement of the thickness distributions of the copper films deposited in each experiment at room temperature, the Si substrates placed parallel to and in front of the target at a distance of 30 mm were masked with a copper mesh of 1.00 ± 0.02 mm aperture and 0.25 ± 0.01 mm wire diameter. The 2D array of ~1.0 mm × 1.0 mm film segments, providing more than 400 height values per sample was mapped using a Veeco Dektak-8 stylus profilometer (Veeco Instruments Inc., Plainview, NY, USA), enabling the determination of the thickness distribution of the films with nanometre resolution. Numerical data were processed and plotted using Origin Pro 9.0 (OriginLab Corporation, Northampton, MA, USA).

In addition to the height data, the profilometry records along the major and minor axes provided information on the surface morphology, specifically the axial dimensions of the particles constituting the films as well. Atomic force microscopy performed with a model PSIA XE-100 (Park Systems, Suwon, Republic of Korea) in the tapping mode completed the size characterization of the building blocks of the film. 

## 3. Results

### 3.1. Analysis of Ablation Conditions: Lack of Contribution of the ns Component to the Ablation Process

Efforts to enhance the energy contrast of the laser system significantly suppressed the contribution of the ns pedestal to the pulse energy. With ASE spot sizes ranging from 0.30 to 3.30 mm^2^, the ASE pulse energy E_ASE_ = 0.22 ± 0.01 mJ yielded a fluence window of 0.037 to 0.0033 Jcm^−2^. To evaluate the potential impact of the ns component on the ablation process, this fluence range was compared to the literature-reported ablation threshold values for copper, which varied depending on the rate of material removal. Low-fluence thresholds of approximately 0.3 Jcm^−2^ [48], 0.5 Jcm^−2^ [49] and about 0.6 Jcm^−2^ [50] were reported for Nd–YAG processing, while high-fluence thresholds ranged from 2.0 Jcm^−2^ to 10 Jcm^−2^ [49,50,51,52]. Of particular relevance to this study is the ablation threshold of 3.4 ± 0.5 Jcm^−2^, derived for KrF excimer laser processing [53,54], which exceeds our maximum ASE fluence of 0.037 Jcm^−2^ calculated for the smallest spot size by two orders of magnitude.

The fact that even the reported low-fluence threshold is almost one order of magnitude higher than our maximum ASE fluence suggests that at 0.037 Jcm^−2^, not even a low-rate ablation characteristic of the low-fluence domain occurs. Therefore, it is unlikely that the ns pedestal causes measurable material removal. Additionally, the maximum intensity of the ASE component was determined to be 4.9 × 10⁶ Wcm^−2^, which was insufficient to induce preplasma or photo-evaporation [55], indicating the interaction of the main pulse with an undisturbed solid-state-like electron density gradient.

Consequently, the ns component of the processing pulse was deemed irrelevant to the ablation process and had no impact on the fs part either, owing to the improved 19:1 energy contrast ratio of the laser system used. As a result, the observed effects were attributed exclusively to the femtosecond part of the pulse. Therefore, we will consider the pulse energy and spot size values of the femtosecond part of the pulse only in the following analysis.

### 3.2. The Clean fs Pulses of Homogeneous Energy Distribution Produce Jet-like Plasma

**Figure 2:**
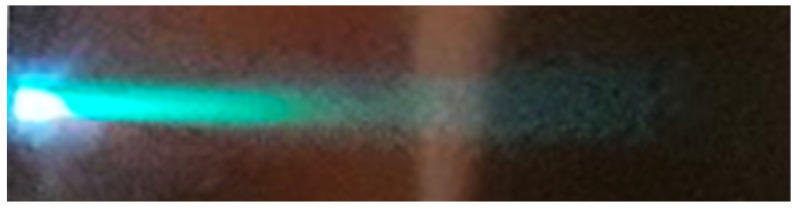
The self-emission of a plasma plume generated by 600 fs pulses of 4.4 ± 0.2 mJ energy hitting the copper target surface as seen by a CCD camera.

The expanding plasma cloud maintained a consistent visual appearance across the range of spot sizes under investigation. Illustrated in Figure 2, the jet-like plume exhibited an extremely forward-directed nature, characterized by aspect ratios around 8. This observation is particularly noteworthy, as it compares favourably even with the highest aspect ratios reported by Harilal et al. from fast gated photography for the ablation of aluminium with 40 fs Ti–sapphire pulses, where a spot diameter of approximately 700 µm, equivalent to 0.38 mm^2^, yielded plume aspect ratios of around 3.7 [46] and approximately 4, as estimated from Figure 6.8 in [56]. To our knowledge, these cited values represent the largest aspect ratios documented within the relevant spot size domain.

### 3.3. The Thickness Distributions of the Deposited Films Are Controlled by the Spot Size

**Figure 3:**
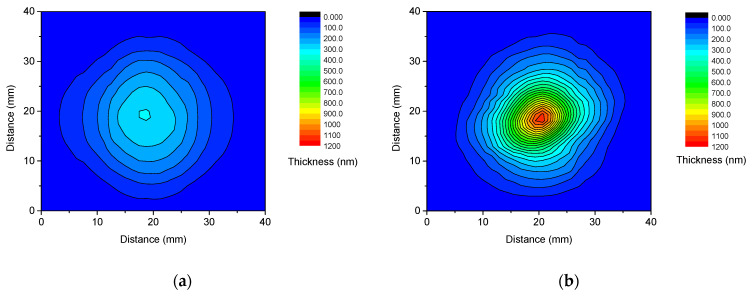
Contour plots of copper films deposited with 600 fs pulses of 4.18 ± 0.19 mJ energy focused to 0.085 mm^2^ (**a**) and 1.01 mm^2^ (**b**) spot areas.

The influence of the spot size on the deposited film characteristics was significant. Contour plots, derived from the measured axial thickness profiles, vividly demonstrate how the film thickness distributions vary as the spot size increases from 0.085 to 1.01 mm^2^. Figure 3 presents the initial and final states of this evolution. The following contrast between these two extremes is remarkable: for the smallest spot size, a flat material distribution was observed with barely discernible ellipticity, in stark contrast to the distribution obtained for the largest spot area, as characterized by pronounced thickness gradients and moderate ellipticity. Maximum thicknesses in the central region of the films exhibited a consistent increase with spot size, initially showing a steep rise followed by a slower rate of increase. Specifically, maximum thicknesses of 305, 664, 824, 970, and 1140 nm were measured for spot areas of 0.085, 0.21, 0.34, 0.59, and 1.01 mm^2^, respectively.

The less apparent ellipticity of the material distributions compared to that of the ablating laser spot is a consequence of the flip-over effect, i.e., the shorter axis of the asymmetric ablating laser spot aligns with the longer axis of the generated plume and thereby with that of the material distribution and vice versa [9,24,26]. Along the longer dimension of the laser spot, a more forward-peaked distribution forms, with greater material concentration in the central region than along the shorter dimension. Conversely, a broadened distribution occurs along the shorter dimension of the spot, resulting in less concentrated material distribution. The differences in the widths of the material distributions and expected maximum film thicknesses along the perpendicular spot axes counterbalance each other, resulting in lower aspect ratios of <1.2 and approximately 1.0 for 1.01 and 0.085 mm^2^ spot areas, respectively, compared to approximately 1.55 characteristic to the spot.

### 3.4. Quantitative Analysis of the Film Thickness Profiles Adopting the f(Θ) = cos^n^Θ Formalism

To quantify the effect of varying spot sizes, we determined the angular distributions of the film thickness along the major and minor axes of the deposited films by fitting the normalized axial film thickness profiles using the cosine formalism [4,10,12,41], ensuring comparison with the literature data presented in the Section 1.

**Figure 4:**
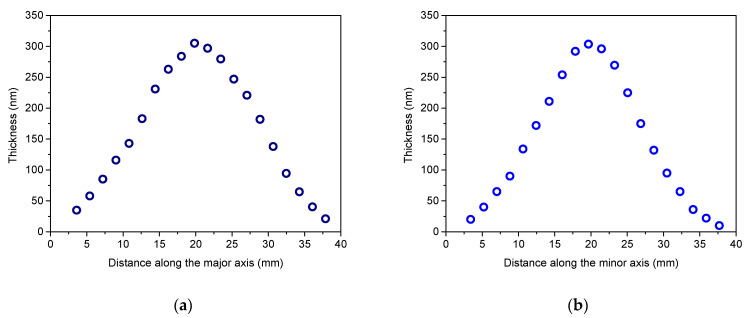
Thickness profiles along the major axis (**a**) and minor axis (**b**) of the copper film deposited with 600 fs pulses of 4.18 ± 0.19 mJ energy focused to a spot area of 0.085 mm^2^.

**Figure 5:**
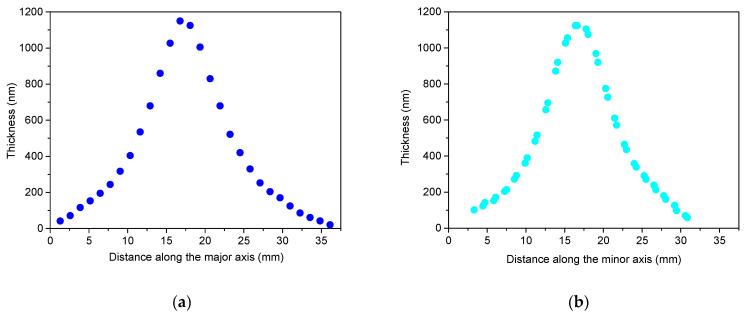
Thickness profiles along the major axis (**a**) and minor axis (**b**) of the copper film deposited with 600 fs pulses of 4.18 ± 0.19 mJ energy focused to a spot area of 1.01 mm^2^.

The axial thickness profiles of the films, represented in Figure 4 for the smallest spot size and in Figure 5 for the largest spot size, reveal notable differences in both maximum thickness and material distribution, particularly in peakedness, between the two films. Specifically, the distribution observed for the irradiated area of 1.01 mm^2^ is more sharply peaked, with a maximum thickness over three times larger than that of the smallest spot size of 0.085 mm^2^, attributed to material concentration near the centre of symmetry. Furthermore, comparing the distributions along the major and minor axes for both spot sizes confirm a reduction in asymmetry compared to the aspect ratio of the laser spot, where the full widths at half maximum for the respective major and minor axes differ by barely more than 10%, with larger asymmetry only observed in the peripheral region, particularly noticeable in the case of the 1.01 mm^2^ spot area.

To facilitate quantitative comparison of the axial film thickness distributions, they were normalised to the maximum thickness, and the horizontal coordinates were centred on the maximum film thickness. In these representations, either single cos*^n^*Θ terms or a linear combination of two such cosinusoidal terms were fitted to the thickness profiles. The shape of the distribution near the peak, i.e., at small angles, does not provide sufficient information for the fitting process, as differences between cos*^n^*Θ terms with various exponents become evident at larger angles. Therefore, relying solely on data measured near the centre is inadequate for a comprehensive analysis. Wide angles exceeding ±30° (based on the target–substrate distance of 30 mm and the 40 mm × 40 mm effective substrate area used for the thickness analysis) enabled us to reliably quantify changes in the lateral distribution as a function of spot size and compare them with relevant results.

**Figure 6:**
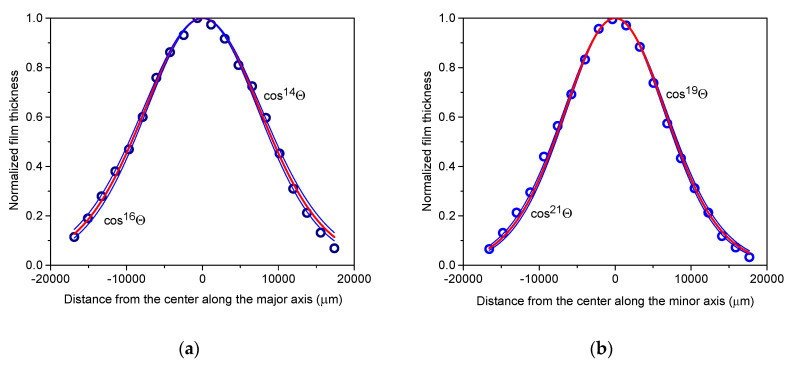
Thickness distributions along the major axis (**a**) and minor axis (**b**) of the copper film deposited using 600 fs pulses of 4.18 ± 0.19 mJ energy focused to a 0.085 mm^2^ spot area. The distribution can be effectively fitted by the single cosine terms cos^15±1^Θ and cos^20±1^Θ (blue lines) for the major and minor axes, respectively. Red lines represent the mean fits.

Within the ±15,000 µm vicinity of the centre, the axial thickness profiles of the film deposited with the smallest spot area of 0.085 mm^2^ can be accurately fitted with single cosine terms of cos^15±1^Θ and cos^20±1^Θ for the major and minor axes, respectively (Figure 6). However, in the peripheral regions beyond ±15,000 µm, the fits slightly deviate from the measured thickness profiles, suggesting that the addition of another cosine term with a low exponent may be necessary to account for the behaviour across the entire area.

**Figure 7:**
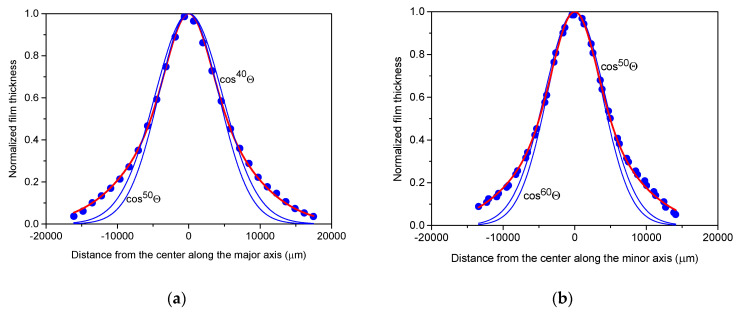
To quantitatively describe the thickness distributions along the major axis (**a**) and minor axis (**b**) of the copper film deposited using 600 fs pulses of 4.18 ± 0.19 mJ energy focused to a 1.01 mm^2^ spot area, two-term cosine-power expressions are required (red lines). Blue lines illustrate inaccurate fits using single cosine terms.

For the major axis of the film deposited at the largest spot area of 1.01 mm^2^, cos^50^Θ provides a perfect fit in the immediate vicinity of the peak (Figure 7). However, beyond distances exceeding ±4000 µm, it progressively diverges from the measured thickness profile. Illustrated by the example of cos^40^Θ, broader functions with lower exponents provide better fits at the wings but show poorer alignment near the peak. Consequently, a single cos^n^Θ function alone cannot adequately fit the measured thickness profile of films deposited at larger spot areas. A successful approach involves introducing fitting functions comprising two cosine terms, namely one with higher exponents, which captures the characteristics of the strongly peaked central domain, and another with smaller exponents, which describes the behaviour of the peripheral material distribution. It is evident that in this scenario, the higher exponent for the major axis should exceed 50. Indeed, combinations such as 0.72cos^60^Θ + 0.28cos^12^Θ, 0.63cos^70^Θ + 0.37cos^14^Θ, and 0.56cos^80^Θ + 0.44cos^16^Θ yielded fits with chi-squared tolerances gradually diminishing to less than 10^−4^ and R^2^ values approaching 0.999, resulting in functions with hardly discernible differences. For the slightly narrower profile along the minor axis, cos^60^Θ was the best fit in the close vicinity of the peak. The approach outlined above resulted in 0.74cos^70^Θ + 0.26cos^11^Θ, 0.66cos^80^Θ + 0.34cos^14^Θ, and 0.59cos^90^Θ + 0.41cos^17^Θ with similar figures of merit.

### 3.5. The Similar Microstructure of the Films Allows for the Comparison of Film Volumes: A Larger Ablation Spot Area Produces a Greater Amount of Deposited Material

**Figure 8:**
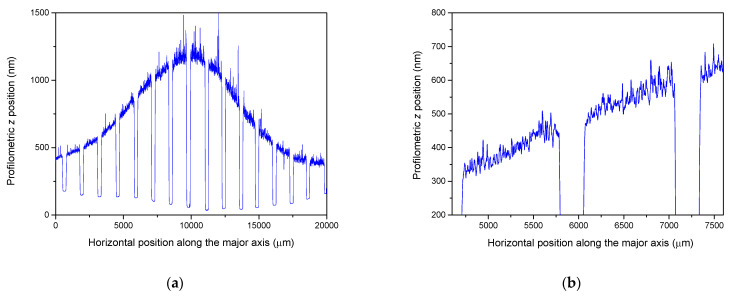
An excerpt from a profilometry scan (**a**) recorded parallel to the major axis of the film deposited with a spot size of 1.01 mm^2^. The magnified portion (**b**) demonstrates the fluctuations due to the surface roughness of the film.

While mapping the sample surfaces along and parallel to the major and minor axes to obtain thickness data, the profilometry records provided abundant information on the grain structure, specifically the axial dimensions of the particles constituting the films (Figure 8).

**Figure 9:**
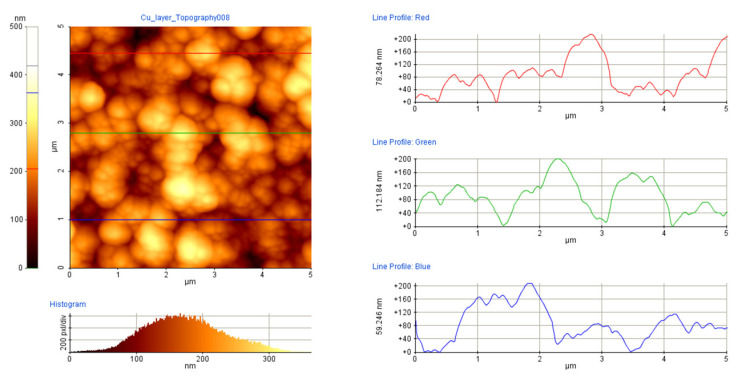
Exemplary atomic force microscopy scan of the film deposited with a spot size of 1.01 mm^2^, revealing the grain structure of the layer.

The film structure is even more discernible in atomic force microscopy images (as exemplified in Figure 9), confirming that all films consisted of particles tens of nanometres thick and hundreds of nanometres in lateral diameter. The consistent microstructure of the films grown at different spot sizes allow for a direct comparison of deposited material volumes. Upon numerically integrating the thickness distributions over the entire area, film volumes of 0.126 mm^3^ and 0.079 mm^3^ were obtained for spot areas of 1.01 mm^2^ and 0.085 mm^2^, respectively. Large-area material processing therefore not only provides highly confined material distribution but also enhanced deposited volume.

## 4. Discussion

The captured self-emission of the plasma plume, depicted in Figure 2 effectively illustrates its jet-like morphology. While the derived plume length-to-width ratio of approximately eight is exceptional, numerical simulations predict even higher ratios for fs plasma phenomena [57]. Considering initial plume thicknesses of tens of nanometres (specifically 20 nm), as estimated from ablation depth figures [58,59], and laser spot major axes ranging from 0.35 to 1.5 mm from our experiments, initial ratios of plume thickness to spot size span between 0.002 mm/0.35 mm and 0.002 mm/1.5 mm, equating to a range between 0.0057 and 0.0013. This theoretical framework indicates that such parameters yield aspect ratios for the expanding plasma plume that exceed even 30.

The apparent independence of the appearance of the self-emitting plasma and its dimensions from the size of the ablating laser spot warrants a more complex analysis. Comparison with the findings by S.S. Harilal clarifies this apparent contradiction. On the one hand, fast gated photography with a 200 ns gate time, capturing images 100 ns after the onset of plasma formation, revealed significant morphological transformations in the plume geometry from spherical to cylindrical symmetry with increasing spot size [43]. On the other hand, ICCD time-integrated images of visible emission from brass plasma, generated by 40 fs pulses of 6 mJ energy on a 100 μm spot size [60,61], revealed that even for this spot size, the visual appearance of the plume remained extremely forward peaked with a length-to-width ratio of approximately four. In these two cases, the behaviour of various plasma components was tracked using two different techniques. While time-integrated photography (similar to that recorded by us and shown in Figure 2) predominantly captured the emission of ionic components, their bright contribution was excluded when using gated photography with sufficient delay. Consequently, the results obtained by these two methodologies lead to the conclusion that while the angular distribution of nanoparticles is strongly influenced by the spot size, the ion flux remains consistently forward peaked regardless of the spot dimensions within the investigated size domain.

The effectiveness of spot size adjustment in controlling the amount of material ablated and deposited on the substrate is clearly demonstrated by the consistently increasing maximum thickness with increasing spot area. Maintaining a constant pulse energy throughout the experiments meant that altering the spot size led to simultaneous modulation of the fluence, where enlarging the spot area from 0.085 to 1.01 mm^2^ resulted in a reduction in energy density from 4.92 to 0.41 Jcm^−2^. The increasing tendency of deposition rates vividly illustrated that increasing the irradiated area not only compensated for, but also suppressed the anticipated significant decline in film growth rate and maximum thickness due to the decreasing fluence.

The potential to enhance the ablation rate by increasing fluence was found to vary across different fluence domains. Slightly above the low-fluence threshold, the pulses practically initiate the removal of only a few atomic layers per pulse from the surface, resulting in low ablation rates that slowly increase with rising fluence. However, upon surpassing the second, higher threshold fluence, the ablation process becomes significantly more efficient, with further increases in fluence leading to steeply escalating ablation rates. Understanding these distinct fluence domains based on previous studies provides valuable context for interpreting our findings.

Amoruso et al. delineated two regions in the ion yield versus fluence plot for a spot size of 0.02 mm^2^ and a P-polarized beam, which was where the low-fluence ion threshold was 0.54 Jcm^−2^, while the high-fluence domain commenced at 0.83 Jcm^−2^ [62]. Given that for pulses shorter than approximately 1 ps, the melting depth was reported to be independent of pulse duration with negligible influence of the initial reflectance [59], it is expected that the thresholds reported exhibit similarity. The reported low-fluence threshold values for Ti–sapphire laser processing, particularly for spot sizes below 10^−2^ mm^2^, scatter between 0.1 and 0.2 Jcm^−2^ [58,59]. In the context of this study, the pioneering comparative analysis of PLD with 500 fs versus 30 ns pulses at 248 nm holds significant importance. A fs ablation threshold of 0.25 ± 0.1 Jcm^−2^ could be derived from Figure 9 in [63].

When focusing 100 fs pulses at 800 nm onto a 35 µm × 35 µm square spot on a copper target surface, Axente et al. distinguished between low- and high-fluence regimes, characterized by gradually and rapidly increasing ablation rates, respectively, based on the fluence dependence of crater depth. A thorough analysis of Figure 3 in [64] reveals two thresholds, approximately at 0.1 Jcm^−2^ and 0.5 Jcm^−2^, respectively. Similarly, two thresholds are apparent in Figure 5 of [65], namely 0.07 Jcm^−2^ and 0.45 Jcm^−2^ for ablation with 500 fs pulses at 800 nm, focused to an elliptical spot of 2140 μm^2^ area. High-fluence thresholds exceeding 0.5 Jcm^−2^ were determined for Ti–sapphire laser ablation by Hermann et al. and Anoop et al. [38,58]. Anoop et al. also reported a more precise value of 0.43 ± 0.08 Jcm^−2^ [39,66]. Micro-machining copper foils with 150 fs pulses at 775 nm in air yielded single-shot and 100-shot ablation thresholds of 0.58 ± 0.05 Jcm^−2^ and 0.55 ± 0.02 Jcm^−2^, respectively, when focusing the Gaussian beam to a spot with a radius of 21.44 ± 0.42 µm [14]. These values are associated with the gentle ablation phase, where the ablation rate was low, with an optical penetration depth derived as 42.7 nm. Strong ablation initiated at 3.19 Jcm^−2^, where the ablation rate was determined by the electron heat diffusion length [67]. A square-shaped spot of 40 µm × 40 µm resulted in a threshold of 0.49 Jcm^−2^ [68]. The successful writing of periodic surface structures into copper with 500 fs pulses at 248 nm, using a fluence of 0.6 Jcm^−2^ [69], along with model calculations [70] and theoretical considerations [18], further affirm a high-fluence threshold of 0.5 Jcm^−2^.

The coherence among the reported thresholds presents a rational and promising overlook. As indicated by the data, gentle ablation of copper with fs pulses typically initiates at around 0.1 Jcm^−2^, with a notable acceleration in the increase in ablation rate from 0.5 Jcm^−2^. This implies that while ablating with spot sizes increasing from 0.085 mm^2^ up to 0.59 mm^2^, we remained within the high-fluence ablation domain, despite the continuous decrease in fluence from 4.92 Jcm^−2^ to 0.71 Jcm^−2^. However, upon further increasing the spot area up to 1.01 mm^−2^, corresponding to 0.41 Jcm^−2^, we transitioned into the low-fluence ablation domain. Considering the fluence dependence of the ablation rate, 4.92 Jcm^−2^, situated in the high-fluence regime, would yield a higher ablation rate, while 0.41 Jcm^−2^, falling below the threshold, would be much less effective in material ablation. Consequently, the increase in spot size from 0.085 mm^−2^ to 1.01 mm^−2^, resulting in a significant decrease in the fluence from 4.92 Jcm^−2^ down to 0.41 Jcm^−2^, should have led to a reduction in the amount of material ablated per pulse and thus in the maximum film thickness. However, contrary to this expectation, we observed the opposite trend.

The resolution to this puzzle lies in the following explanation. The observed trend regarding the spot size dependence of maximum thickness arises from an interplay between the narrowing angular distribution of ejecta, resulting from increased irradiated areas, and the reduction in removal rate, due to decreased fluence. Our findings unmistakably demonstrate that within a fluence domain nearing the ablation threshold, controlling the spot size is far more effective in regulating maximum thickness than energy control at fixed ablated area. This observation reaffirms that it is the combination of pulse energy and spot size, rather than fluence alone, that determines the outcome of the ablation process. Evidently, beyond a spot size, the material confinement can no longer surpass the decrease in ablation rate, resulting in less effective film deposition. Further increase in spot size leads to reaching the ablation threshold. In our case, this transition likely occurs around 4 mm^2^, given a pulse energy of 4.18 mJ. Although our results may not be readily comparable to relevant data due to limited studies with high-energy lasers, maintaining constant laser energy while altering the laser spot size, one study reinforces the validity of such a trend, where Jegenyés et al. reported an increase in maximum thickness with increasing spot size while varying laser intensity from 13 × 10^12^ W/cm^2^ down to 7.4 × 10^12^ W/cm^2^ when depositing diamond-like carbon films with a similar laser setup [71]. In a subsequent experiment using a Ti–sapphire laser with lower energy pulses, a critical intensity of 4 × 10^12^ W/cm^2^ was identified, beyond which further increases in spot size led to a decrease in peak film thickness.

The comparison of the contour plots depicted in Figure 3 offers a clear visual representation of how the material distribution evolves as the irradiated area expands from 0.085 mm^2^ to 1.01 mm^2^. While numerous studies have investigated the variations in plasma shape and film morphology with spot size [30,60,61], the work of R. K. Singh provided specific insight. They highlighted that for spot sizes of ≤100 µm in diameter, the geometry of the expanding plasma tended to be spherical. However, a significant transition occurs between 100 µm and 8 mm, leading to a shift towards a one-dimensional geometry with minimal lateral expansion when the laser spot size reaches 8 mm. Although the paper does not explicitly specify the pulse duration domain to which the results pertain, an inference from the publication date suggests it likely involves nanosecond pulses. [7] Our spot areas fall within the lower half of this critical size domain, indicating that we did not reach the predicted one-dimensional limit. Nonetheless, as depicted in Figure 1, at least the ionic components of the plume exhibited a practically one-dimensional shape, accompanied by a strongly peaked angular distribution of the deposited material. As fluence increases, the forward-directed nature of the plasma and the confined distribution of the resulting film become more pronounced. Therefore, besides enlarging the irradiated area, increasing fluence is an additional approach to address one-dimensional plume geometry with even higher aspect ratios.

To quantitatively assess the differences between the thickness distributions derived for different PLD parameters and for comparative analyses, two widely accepted approaches are employed in the PLD community, specifically the f(Θ) = cos^n^Θ [10,24] and f(Θ) ~ k^2^/z^2^ (1 + k^2^tan^2^Θ)^−3/2^ [72,73,74] formalisms. Here, Θ denotes the radial angle, defined as Θ = arctan(*r*/*z*), with *r* representing the radial coordinate (i.e., the distance from the centre of symmetry on the substrate laterally) and *z* indicating the target-to-substrate distance. The exponents *n* and *k*, referred to as forward peaking factors, express the narrowness of the distribution. Both formalisms offer simplified approaches for small angles and are effective for angles achievable in PLD.

However, an inherent challenge with assessments based on fitting with either of the aforementioned formulas is that deviations between the measured angular distributions and the model functions used are particularly noticeable at large angles [75]. The f(Θ) = cos^n^Θ approach offers an advantage in its capability to fit a broader range of the distributions, including their wings, adequately through the linear combination of two cosinusoidal terms with different exponents. In this study, we employed this formalism to quantify the differences in large-area thickness distributions obtained for the two spot sizes. Evidently, this methodology allowed us to consider the literature data only where a similar cosinusoidal approach was employed.

When ablating with ns lasers, angular distributions with exponents as high as 130 have been documented for the ion flux [76], while the majority of film thickness profiles were characterized by exponents ranging from 4 to 40 [10,21]. Particularly elevated values were observed for excimer laser processing, where the combination of high pulse energies enabling large spot sizes and a flat top beam profile resulted in exponents up to 61 for the more peaked component of the bifunctional formalism [11,77,78,79,80]. Although the influence of the wavelength of the ablating laser could not be reliably isolated from changes in other parameters, in the ultraviolet regime, an increase in spot size generally led to a notable sharpening of the plasma plume geometry [81].

Given the significantly distinct ablation mechanisms operating across fluence domains and spanning several orders of magnitudes, direct comparison of individual fs versus ns data poses challenges. Nevertheless, insights from a select comparative studies, where a primary process parameter such as fluence or irradiated area was held constant and demonstrate a consistent trend, namely fs PLD consistently yields sharper film thickness distributions. For instance, when ablating aluminium targets with a Ti–sapphire laser (40 fs at 800 nm) and a Nd–YAG laser (6 ns at 1064 nm) while maintaining the same fluence by focusing the pulses of 6 mJ energy onto a spot of approximately 1 × 10^3^ µm^2^ on the target surface, Verhoff et al. estimated that the full width at half maximum of the ion flux was approximately three times lower for fs processing compared to ns processing [82]. The data presented in Figure 5 in [82] convincingly demonstrated that the angular dependence of mass deposition profiles were much more peaked for the fs plume compared to the ns plume. Similarly, the thickness distribution of diamond-like carbon films prepared by ablating approximately 5 × 10^−2^ mm^2^ target areas with 500 fs versus 30 ns pulses at 248 nm, proved to be more narrowed for fs ablation [54]. In terms of cos*^n^*Θ, *n* increased from approximately 8 to 12 with a concomitant increase in peak thickness. The increase in the impact area resulted in narrowing in both cases, but with different effectiveness, such that the change induced by the same alteration in spot size was much less pronounced for ns processing.

For the assessment of deposition efficiency, the determination of the angular distribution of the material is essential. For spot areas of 0.085 mm^2^ and 1.01 mm^2^, the main components of the angular distribution of the film material deposited exhibited exponents of *k* = 15 ± 1 and >50, respectively, in terms of the representation *f*(Θ) = *A*cos*^k^*Θ + (1 − *A*)cos*^p^*Θ. The increase in exponents with increasing spot size indicates a greater degree of narrowing. While considering that fs PLD typically yields films with narrower material distributions compared to ns PLD, the exponents exceeding 60 derived in our case are particularly noteworthy. A critical analysis of the relevant literature data suggests that the underlying cause of this phenomenon is that excimer laser processing, i.e., ablation in the ultraviolet range with pulses possessing energy profiles more homogeneous than Gaussian, is beneficial. In our study, the unique characteristics of the laser emitting temporally clean sub-ps pulses at 248 nm, accompanied by a perfectly homogeneous beam profile undoubtedly contributed to the exceptional performance observed. As highlighted in the Section 3, the material flow predominantly consisted of nanoparticles. It is widely acknowledged that ultrashort laser ablation produces nanoparticles. Our findings suggest that material flows are composed primarily of nanoparticles yield films with a more confined thickness profiles compared to those containing neutrals (atomic components).

## 5. Conclusions

Focusing the beam of the updated Szatmári-type hybrid dye-excimer laser system emitting 248 nm fs pulses with a maximum energy of 20 mJ to varying areas between 0.085 mm^2^ and 1.01 mm^2^ on copper, while maintaining constant pulse energy, revealed a significant impact on the material distribution of both the plasma plume and the deposited films. The axial thickness profiles of the films could be perfectly described using the *f*(Θ) = *A*cos*^k^*Θ + (1 − *A*)cos*^p^*Θ formalism, with exponents exceeding 50. As the spot size approached its largest, an extremely forward-peaked material flow was observed, resulting in films with material distributions highly confined in the close vicinity of the symmetry centre.

Two primary factors emerged as contributors to this highly localized material deposition. Firstly, the unique characteristics of the laser system, specifically, the temporally clean 600 fs pulses with 4.18 ± 0.19 mJ energy, providing homogeneously irradiated spots on the target surface. Secondly, by adjusting the dimensions of the ablating laser spot, the spatial distribution of the plasma plume could be effectively tuned. The observed tendencies suggest that further increases in spot size could culminate in a plasma geometry characterized by a cylindrical material flow perpendicular to the substrate, mirroring in its cross-sectional shape that of the ablating laser spot. This would enable a nearly perfectly localized film deposition, with the covered area and growth rates scaling with pulse energy. The availability of joule-class femtosecond lasers thereby enables the depositing films of exotic materials onto localized areas spanning several cm^2^.

Additionally, our findings underscore the intricate interplay between the effect of changes in laser-irradiated area and laser pulse energy on plasma plume dynamics and film characteristics. Ongoing systematic studies aim to further elucidate the extent to which the unique properties of the laser system influence the dependence of thickness distribution and deposition rate on spot size, as well as to explore the potential extreme values that can be reached regarding process and film parameters.

## Data Availability

The data presented in this study are available on request from Tamás Szörényi, t.szorenyi@physx.u-szeged.hu.

## References

[1] Singh R.K., Holland O.W., Narayan J. (1990). Theoretical Model for Deposition of Superconducting Thin Films Using Pulsed Laser Evaporation Technique. J. Appl. Phys..

[2] Singh R.K., Biunno N., Narayan J. (1988). Microstructural and Compositional Variations in Laser-Deposited Superconducting Thin Films. Appl. Phys. Lett..

[3] Singh R.K., Narayan J. (1990). Pulsed-Laser Evaporation Technique for Deposition of Thin Films: Physics and Theoretical Model. Phys. Rev. B.

[4] Kools J.C.S., Van De Riet E., Dieleman J. (1993). A Simple Formalism for the Prediction of Angular Distributions in Laser Ablation Deposition. Appl. Surf. Sci..

[5] Gorbunov A.A., Pompe W., Sewing A., Gaponov S.V., Akhsakhalyan A.D., Zabrodin I.G., Kas’kov I.A., Klyenkov E.B., Morozov A.P., Salaschenko N.N. (1996). Ultrathin Film Deposition by Pulsed Laser Ablation Using Crossed Beams. Appl. Surf. Sci..

[6] Saenger K.L. (1991). On the Origin of Spatial Nonuniformities in the Composition of Pulsed-Laser-Deposited Films. J. Appl. Phys..

[7] Singh R.K. (1997). Spatial Thickness Variations in Laser-Deposited Thin Films. Mater. Sci. Eng. B.

[8] Santagata A., Marotta V., D’Alessio L., Teghil R., Ferro D., DeMaria G. (1997). Study of the Gaseous Phase from Pulsed Laser Ablation of Titanium Carbide. Appl. Surf. Sci..

[9] Mele A., Guidoni A.G., Kelly R., Miotello A., Orlando S., Teghil R., Flamini C. (1996). Angular Distribution and Expansion of Laser Ablation Plumes Measured by Fast Intensified Charge Coupled Device Photographs. Nucl. Instrum. Methods Phys. Res. Sect. B Beam Interact. Mater. At..

[10] Saenger K.L. (1994). Angular Distribution of Ablated Material. Pulsed Laser Deposition of Thin Films.

[11] Venkatesan T., Wu X.D., Inam A., Wachtman J.B. (1988). Observation of Two Distinct Components during Pulsed Laser Deposition of High *T c* Superconducting Films. Appl. Phys. Lett..

[12] Weaver I., Lewis C.L.S. (1996). Polar Distribution of Ablated Atomic Material during the Pulsed Laser Deposition of Cu in Vacuum: Dependence on Focused Laser Spot Size and Power Density. J. Appl. Phys..

[13] Neifeld R.A., Gunapala S., Liang C., Shaheen S.A., Croft M., Price J., Simons D., Hill W.T. (1988). Systematics of Thin Films Formed by Excimer Laser Ablation: Results on SmBa_2_Cu_3_O_7_. Appl. Phys. Lett..

[14] Thum-Jager A., Rohr K. (1999). Angular Emission Distributions of Neutrals and Ions in Laser Ablated Particle Beams. J. Phys. D Appl. Phys..

[15] Doggett B., Lunney J.G. (2011). Expansion Dynamics of Laser Produced Plasma. J. Appl. Phys..

[16] Thum A., Rupp A., Rohr K. (1994). Two-Component Structure in the Angular Emission of a Laser-Produced Ta Plasma. J. Phys. D Appl. Phys..

[17] Mann K., Rohr K. (1992). Differential Measurement of the Absolute Ion Yield from Laser-Produced C Plasmas. Laser Part. Beams.

[18] Konomi I., Motohiro T., Asaoka T. (2009). Angular Distribution of Atoms Ejected by Laser Ablation of Different Metals. J. Appl. Phys..

[19] Torrisi L., Andò L., Ciavola G., Gammino S., Barnà A. (2001). Angular Distribution of Ejected Atoms from Nd:YAG Laser Irradiating Metals. Rev. Sci. Instrum..

[20] Thestrup B., Toftmann B., Schou J., Doggett B., Lunney J.G. (2002). Ion Dynamics in Laser Ablation Plumes from Selected Metals at 355 Nm. Appl. Surf. Sci..

[21] Del Coso R., Perea A., Serna R., Chaos J.A., Gonzalo J., Solis J. (1999). Critical Parameters Influencing the Material Distribution Produced by Pulsed Laser Deposition. Appl. Phys. A Mater. Sci. Process..

[22] Pietsch W. (1996). Effect of Knudsen-Layer Formation on the Initial Expansion and Angular Distribution of a Laser-Produced Copper Plasma at Reduced Pressure of Air. J. Appl. Phys..

[23] Szörényi T., Ballesteros J.M. (1997). Dependence of the Thickness Profile of Pulsed Laser Deposited Bismuth Films on Process Parameters. Appl. Surf. Sci..

[24] Guidoni A.G., Kelly R., Mele A., Miotello A. (1997). Heating Effects and Gas-Dynamic Expansion of the Plasma Plume Produced by Irradiating a Solid with Laser Pulses. Plasma Sources Sci. Technol..

[25] Kelly R., Dreyfus R.W. (1988). Reconsidering the mechanisms of laser sputtering with Knudsen-layer formation taken into account. Nucl. Instrum. Methods Phys. Res. Sect. B Beam Interact. Mater. At..

[26] Mele A., Giardini Guidoni A., Kelly R., Miotello A., Orlando S., Teghil R. (1996). Spatial Distribution of Laser-Ablated Material by Probing a Plasma Plume in Three Dimensions. Appl. Surf. Sci..

[27] Schneider C.W., Lippert T. (2023). PLD Plasma Plume Analysis: A Summary of the PSI Contribution. Appl. Phys. A.

[28] Haider A.J., Alawsi T., Haider M.J., Taha B.A., Marhoon H.A. (2022). A Comprehensive Review on Pulsed Laser Deposition Technique to Effective Nanostructure Production: Trends and Challenges. Opt. Quant. Electron..

[29] Shepelin N.A., Tehrani Z.P., Ohannessian N., Schneider C.W., Pergolesi D., Lippert T. (2023). A Practical Guide to Pulsed Laser Deposition. Chem. Soc. Rev..

[30] Harilal S.S. (2007). Influence of Spot Size on Propagation Dynamics of Laser-Produced Tin Plasma. J. Appl. Phys..

[31] Haverkamp J., Mayo R.M., Bourham M.A., Narayan J., Jin C., Duscher G. (2003). Plasma Plume Characteristics and Properties of Pulsed Laser Deposited Diamond-like Carbon Films. J. Appl. Phys..

[32] Friichtenicht J.F., Utterback N.G., Valles J.R. (1976). Intense Accelerated Metal Ion Beam Utilizing Laser Blowoff. Rev. Sci. Instrum..

[33] Tyunina M., Wittborn J., Björmander C., Rao K.V. (1998). Thickness Distribution in Pulsed Laser Deposited PZT Films. J. Vac. Sci. Technol. A Vac. Surf. Film..

[34] De Bonis A., Galasso A., Latini A., Rau J.V., Santagata A., Curcio M., Teghil R. (2019). Femtosecond Pulsed Laser Deposition of Chromium Diboride-Rich Thin Films. Coatings.

[35] Amoruso S., Bruzzese R., Wang X., Nedialkov N.N., Atanasov P.A. (2007). Femtosecond Laser Ablation of Nickel in Vacuum. J. Phys. D Appl. Phys..

[36] Donnelly T., Lunney J.G., Amoruso S., Bruzzese R., Wang X., Ni X. (2010). Dynamics of the Plumes Produced by Ultrafast Laser Ablation of Metals. J. Appl. Phys..

[37] Toftmann B., Doggett B., Budtz-Jørgensen C., Schou J., Lunney J.G. (2013). Femtosecond Ultraviolet Laser Ablation of Silver and Comparison with Nanosecond Ablation. J. Appl. Phys..

[38] Anoop K.K., Polek M.P., Bruzzese R., Amoruso S., Harilal S.S. (2015). Multidiagnostic Analysis of Ion Dynamics in Ultrafast Laser Ablation of Metals over a Large Fluence Range. J. Appl. Phys..

[39] Anoop K.K., Ni X., Wang X., Amoruso S., Bruzzese R. (2014). Fast Ion Generation in Femtosecond Laser Ablation of a Metallic Target at Moderate Laser Intensity. Laser Phys..

[40] Li N., Ni X., Hong R., Donnelly T., Wang X., Amoruso S., Wang C. The Spatial Detection on Distribution of Metal Nano-Particles during Femtosecond Laser Ablation. Proceedings of the International Symposium on Photoelectronic Detection and Imaging 2009.

[41] Donnelly T., Lunney J.G., Amoruso S., Bruzzese R., Wang X., Ni X. (2010). Angular Distributions of Plume Components in Ultrafast Laser Ablation of Metal Targets. Appl. Phys. A.

[42] Donnelly T., Lunney J.G., Amoruso S., Bruzzese R., Wang X., Phipps C. (2010). Plume Dynamics in Femtosecond Laser Ablation of Metals. AIP Conf. Proc..

[43] Harilal S.S., Diwakar P.K., Polek M.P., Phillips M.C. (2015). Morphological Changes in Ultrafast Laser Ablation Plumes with Varying Spot Size. Opt. Express.

[44] Garrelie F., Loir A.S., Donnet C., Rogemond F., Le Harzic R., Belin M., Audouard E., Laporte P. (2003). Femtosecond Pulsed Laser Deposition of Diamond-like Carbon Thin Films for Tribological Applications. Surf. Coat. Technol..

[45] Loir A.S., Garrelie F., Donnet C., Rogemond F., Subtil J.L., Forest B., Belin M., Laporte P. (2004). Towards the Deposition of Tetrahedral Diamond-like Carbon Films on Hip Joints by Femtosecond Pulsed Laser Ablation. Surf. Coat. Technol..

[46] Szatmári S., Schäfer F.P., Müller-Horsche E., Müchenheim W. (1987). Hybrid dye-excimer laser system for the generation of 80 fs, 900 GW pulses at 248 nm. Opt. Commun..

[47] Gilicze B., Barna A., Kovács Z., Szatmári S., Földes I.B. (2016). Plasma Mirrors for Short Pulse KrF Lasers. Rev. Sci. Instrum..

[48] Jordan R., Cole D., Lunney J.G., Mackay K., Givord D. (1995). Pulsed Laser Ablation of Copper. Appl. Surf. Sci..

[49] Shin B.S., Oh J.Y., Sohn H. (2007). Theoretical and Experimental Investigations into Laser Ablation of Polyimide and Copper Films with 355-Nm Nd:YVO4 Laser. J. Mater. Process. Technol..

[50] Tunna L., Kearns A., O’Neill W., Sutcliffe C.J. (2001). Micromachining of Copper Using Nd:YAG Laser Radiation at 1064, 532, and 355 Nm Wavelengths. Opt. Laser Technol..

[51] Zhang W., Yao Y.L., Chen K. (2001). Modelling and Analysis of UV Laser Micromachining of Copper. Int. J. Adv. Manuf. Technol..

[52] Tang G., Abdolvand A. (2011). Laser-Assisted Highly Organized Structuring of Copper. Opt. Mater. Express.

[53] Lorusso A., Nassisi V., Buccolieri A., Buccolieri G., Castellano A., Leo L.S., Di Giulio M., Torrisi L., Caridi F., Borrielli A. (2008). Laser Ablation Threshold of Cultural Heritage Metals. Radiat. Eff. Defects Solids.

[54] Mueller F., Mann K.R., Simon P., Bernstein J.S., Zaal G.J. Comparative Study of Deposition of Thin Films by Laser-Induced PVD with Femtosecond and Nanosecond Laser Pulses. Proceedings of the OE/LASE’93: Optics, Electro-Optics, and Laser Applications in Scienceand Engineering.

[55] Földes I.B., Gál K., Jhász Z., Kedves M.Á., Kocsis G., Szatmári S., Veres G. (2000). Properties of High Harmonics Generated by Ultrashort UV Laser Pulses on Solid Surfaces. Laser Phys..

[56] Harilal S.S., Freeman J.R., Diwakar P.K., Hassanein A., Musazzi S., Perini U. (2014). Femtosecond Laser Ablation: Fundamentals and Applications. Laser-Induced Breakdown Spectroscopy.

[57] Komashko A., Feit M., Rubenchik A. Modeling of Long-Term Behavior of Ablation Plumes Produced with Ultrashort Laser Pulses. Proceedings of the SPIE—The International Society for Optical Engineering.

[58] Hermann J., Noël S., Itina T.E., Axente E., Povarnitsyn M.E. (2008). Correlation between Ablation Efficiency and Nanoparticle Generation during the Short-Pulse Laser Ablation of Metals. Laser Phys..

[59] Byskov-Nielsen J., Savolainen J.-M., Christensen M.S., Balling P. (2011). Ultra-Short Pulse Laser Ablation of Copper, Silver and Tungsten: Experimental Data and Two-Temperature Model Simulations. Appl. Phys. A.

[60] Freeman J.R., Harilal S.S., Diwakar P.K., Verhoff B., Hassanein A. (2013). Comparison of Optical Emission from Nanosecond and Femtosecond Laser Produced Plasma in Atmosphere and Vacuum Conditions. Spectrochim. Acta Part B At. Spectrosc..

[61] Verhoff B., Harilal S.S., Freeman J.R., Diwakar P.K., Hassanein A. (2012). Dynamics of Femto- and Nanosecond Laser Ablation Plumes Investigated Using Optical Emission Spectroscopy. J. Appl. Phys..

[62] Amoruso S., Wang X., Altucci C., De Lisio C., Armenante M., Bruzzese R., Velotta R. (2000). Thermal and Nonthermal Ion Emission during High-Fluence Femtosecond Laser Ablation of Metallic Targets. Appl. Phys. Lett..

[63] Preuss S., Demchuk A., Stuke M. (1995). Sub-Picosecond UV Laser Ablation of Metals. Appl. Phys. A.

[64] Axente E., Noël S., Hermann J., Sentis M., Mihailescu I.N. (2009). Subpicosecond Laser Ablation of Copper and Fused Silica: Initiation Threshold and Plasma Expansion. Appl. Surf. Sci..

[65] Hashida M., Semerok A.F., Gobert O., Petite G., Izawa Y., Wagner J.F. (2002). Ablation Threshold Dependence on Pulse Duration for Copper. Appl. Surf. Sci..

[66] Ni X., Anoop K.K., Wang X., Paparo D., Amoruso S., Bruzzese R. (2014). Dynamics of Femtosecond Laser-Produced Plasma Ions. Appl. Phys. A.

[67] Mannion P.T., Magee J., Coyne E., O’Connor G.M., Glynn T.J. (2004). The Effect of Damage Accumulation Behaviour on Ablation Thresholds and Damage Morphology in Ultrafast Laser Micro-Machining of Common Metals in Air. Appl. Surf. Sci..

[68] Momma C., Nolte S., Chichkov B.N., Alvensleben F.V., Tünnermann A. (1997). Precise Laser Ablation with Ultrashort Pulses. Appl. Surf. Sci..

[69] Simon P., Ihlemann J. (1997). Ablation of Submicron Structures on Metals and Semiconductors by Femtosecond UV-Laser Pulses. Appl. Surf. Sci..

[70] Cheng C.W., Wang S.Y., Chang K.P., Chen J.K. (2016). Femtosecond Laser Ablation of Copper at High Laser Fluence: Modeling and Experimental Comparison. Appl. Surf. Sci..

[71] Jegenyes N., Toth Z., Hopp B., Klebniczki J., Bor Z., Fotakis C. (2006). Femtosecond Pulsed Laser Deposition of Diamond-like Carbon Films: The Effect of Double Laser Pulses. Appl. Surf. Sci..

[72] Anisimov S.I., Bäuerle D., Luk’yanchuk B.S. (1993). Gas Dynamics and Film Profiles in Pulsed-Laser Deposition of Materials. Phys. Rev. B.

[73] Anisimov S.I., Luk’yanchuk B.S., Luches A. (1996). An Analytical Model for Three-Dimensional Laser Plume Expansion into Vacuum in Hydrodynamic Regime. Appl. Surf. Sci..

[74] Cachoncinlle C., Millon E., Portier X., Hebert C., Perrière J., Nistor M. (2022). Anisotropy of Physical Properties in Pulsed Laser-Deposited ZnO Films. Appl. Phys. A.

[75] Pryds N., Schou J., Linderoth S. (2007). The Spatial Thickness Distribution of Metal Films Produced by Large Area Pulsed Laser Deposition. Appl. Surf. Sci..

[76] Champeaux C., Damiani D., Aubreton J., Catherinot A. (1993). Mass Spectrometric Investigation of the KrF Laser-Induced Plasma Plume Created above an YBaCuO Superconducting Target: Correlation with Thickness Distribution of Deposited Thin Films. Appl. Surf. Sci..

[77] Wolf P.J., Christensen T.M., Coit N.G., Swinford R.W. (1993). Thin Film Properties of Germanium Oxide Synthesized by Pulsed Laser Sputtering in Vacuum and Oxygen Environments. J. Vac. Sci. Technol. A Vac. Surf. Film..

[78] Muenchausen R.E., Hubbard K.M., Foltyn S., Estler R.C., Nogar N.S., Jenkins C. (1990). Effects of Beam Parameters on Excimer Laser Deposition of YBa_2_Cu_3_O_7−δ_. Appl. Phys. Lett..

[79] Afonso C.N., Serna R., Catalina F., Bermejo D. (1990). Good-Quality Ge Films Grown by Excimer Laser Deposition. Appl. Surf. Sci..

[80] Pavlišta M., Hrdlička M., Němec P., Přikryl J., Frumar M. (2008). Thickness Distribution of Thin Amorphous Chalcogenide Films Prepared by Pulsed Laser Deposition. Appl. Phys. A.

[81] Torrisi L., Gammino S., Andò L., Nassisi V., Doria D., Pedone A. (2003). Comparison of Nanosecond Laser Ablation at 1064 and 308 Nm Wavelength. Appl. Surf. Sci..

[82] Verhoff B., Harilal S.S., Hassanein A. (2012). Angular Emission of Ions and Mass Deposition from Femtosecond and Nanosecond Laser-Produced Plasmas. J. Appl. Phys..

